# Improper lung volume-dose parameters are risk factors for acute fatal radiation pneumonitis among esophageal cancer patients receiving chemoradiotherapy: a case-control study

**DOI:** 10.3389/fonc.2025.1535676

**Published:** 2025-01-28

**Authors:** Ruinuo Jia, Liuyan Li, Xiaoyi Liu, Junqian Zhang, Shuoguo Li, Ziqi Wang, Jingxia Li, Bingyi Xu, Manxi Sheng, Lei Ni, Danni Yang, Shegan Gao

**Affiliations:** ^1^ Cancer Hospital, The First Affiliated Hospital (College of Clinical Medicine) of Henan University of Science and Technology, Luoyang, China; ^2^ Henan Key Laboratory of Microbiome and Esophageal Cancer Prevention and Treatment, Luoyang, China; ^3^ Henan Key Laboratory of Cancer Epigenetics, Luoyang, China

**Keywords:** acute fatal radiation pneumonitis, esophageal cancer, chemoradiotherapy, lung volume-dose parameters, propensity score matching

## Abstract

**Background:**

Definitive concurrent chemoradiotherapy (DCRT) is the standard treatment for locally advanced unresectable esophageal cancer (EC). However, acute fatal radiation pneumonitis (AFRP) is one of the most harmful complications and it is still controversial which factors pose a greater risk.

**Aim:**

This case-control study aims to investigate the relationship between AFRP and lung volume-dose parameters in patients with esophageal cancer undergoing DCRT.

**Methods:**

Cases are patients who died of AFRP after DCRT, whereas controls are patients who did not develop RP. Participants were enrolled using the International Classification of Diseases Codes Searching and then verified by medical record review. One-to-three propensity score matching was performed between EC patients undergoing DCRT who died from AFRP and those who did not develop radiation pneumonitis(RP). Prognostic factors were determined using univariate and multivariate analyses. The exposure factors were lung volume-dose parameters, including V5, V20, V30, and mean lung dose (MLD). Overall survival was compared between the two groups of patients before and after propensity score matching.

**Results:**

17 cases were confirmed with AFRP among 568 EC patients were treated with DCRT between June 2008 and June 2013, and 51 cases with no RP matched by PSM method in the control group. The median V5 and MLD values in the case group were significantly higher than the control group: 88.39% versus 65.045% and 17.325 gray (Gy) versus 14.38 Gy, respectively. V5 > 60%, V20 > 25%, and MLD > 15 Gy were identified as independent risk factors for AFRP. V5 > 80% significantly increased the susceptibility to AFRP and predicted worse overall survival.

**Conclusion:**

V5 > 60%, V20 > 25% and MLD > 15 Gy are key risk factors for AFRP in EC patients undergoing DCRT. Furthermore, V5 > 80% is a strong indicator of mortality risk.

## Introduction

Esophageal cancer (EC) is a prevalent and highly lethal malignancy worldwide. According to GLOBOCAN 2020 data, there were approximately 604,100 new cases and 544,076 cancer-related deaths from EC in 2020. The incidence of EC is higher in males, with a ratio of 2-3 times compared to females (1). Esophageal squamous cell carcinoma (ESCC) is the predominant histological type in China, while esophageal adenocarcinoma is more common in Europe and the United States. Although the incidence of ESCC in China has declined in recent years, it remains the mainly prevalent malignant disease. EC is the fourth leading cause of cancer- related mortality among men in China, accounting for 9.8% of all cancer deaths annually (2, 3). Locally advanced disease is present in approximately half of all EC cases worldwide, with a dismal 5-year survival rate of less than 20% (4).

Traditionally, radical esophagectomy is performed for surgically resectable tumors, while radiotherapy is employed for locally advanced, unresectable tumors. However, radiation therapy as a stand-alone treatment yields unsatisfactory long-term survival outcomes. Concurrent chemoradiotherapy (CRT) with cisplatin-based and 5-fluorouracil (5-FU)-based chemotherapy has demonstrated superior efficacy compared to radiotherapy alone (5–9). Meanwhile, compared with radiation therapy alone or sequential chemoradiotherapy, CRT is associated with increased grade 3 toxicity and side effects, including radiation pneumonitis (RP), esophagitis, and cardiac toxicity (10, 11).

For EC patients who received CRT, the treatment-related acute toxicity results from biological effects on rapidly dividing cells within the irradiated volume, including the lungs, esophagogastric mucosa, and heart. RP, a well-known complication of thoracic radiation therapy for malignant diseases, can result in pulmonary interstitial fibrosis, characterized by diffuse irregular interlobular thickness and honeycombing on CT images, which indicates poor prognosis (12, 13). Risk factors for RP are thought to stem from both radiation-related factors and patient condition, such as poor performance status, elderly age, and pre- existing lung conditions (14).

Advancements in radiotherapy techniques, such as stereotactic body radiation therapy (SBRT) and proton beam therapy (PBT), have resulted in a decline in RP incidence and rare occurrences of fatal RP (15–18). However, there are different reasons why SBRT and PBT cannot be used for esophageal cancer. The former is not applicable because it is necessary to irradiate a certain area from the primary site when treating esophageal cancer, and the latter is an issue whether the facility has the equipment. The three-dimensional conformal radiation therapy (3DCRT) and intensity-modulated radiation therapy (IMRT) using linear accelerators being the most commonly used radiation modalities. Therefore, it is crucial to identify strategies to prevent serious RP when combining these techniques with chemotherapy for EC patients.

Inappropriate lung dosimetric parameters have been reported to contribute to the development of RP (19, 20). In a previous study, we identified certain serum biomarkers as predictive factors for RP among EC patients (21). However, there is still controversial regarding the most significant risk factor for RP: a larger volume receiving a low radiation dose or a smaller volume receiving a high radiation dose (22, 23). 17 cases of acute fatal radiation pneumonitis (AFRP) among 568 patients who underwent definitive concurrent chemoradiotherapy (DCRT) for EC at our site were observed. The substantial treatment-related mortality associated with EC presents an ideal model to investigate the high- risk factors for AFRP. Therefore, we conducted a retrospective case-control study utilizing medical history data, employing propensity score matching (PSM), to investigate the most prominent and lethal risk factor for AFRP in the EC population treated with DCRT.

## Materials and methods

### Study design

**Figure 1:**
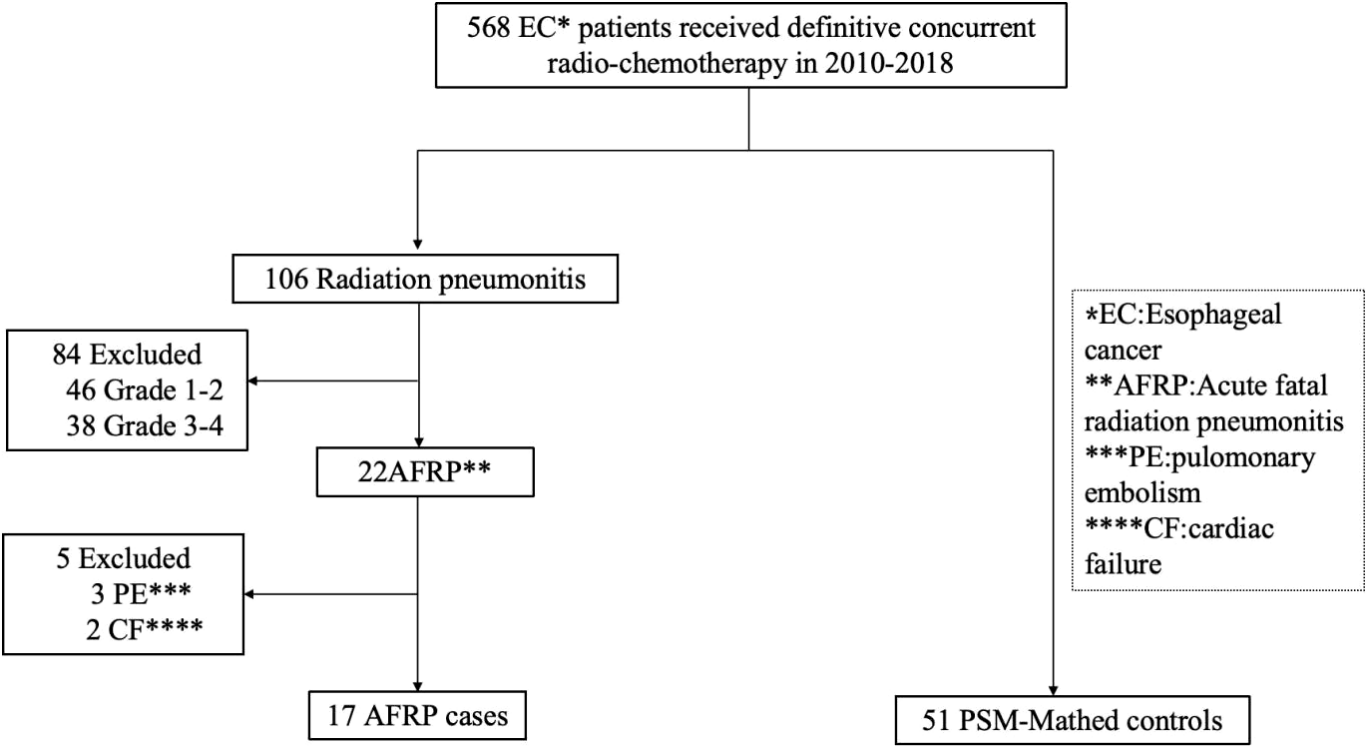
Flow diagram showing the patients recruitment process. *EC, Esophageal cancer; **AFRP, Acute fatal radiation pneumonitis; ***PE, pulomonary embolism; ****CF, cardiac failure.

This retrospective case-control study utilized patient records and medical data and employed one-to-three PSM matching. The patient selection process is shown in **Figure 1**. The radiation delivery methods, computed tomography (CT) scans and X- ray images, treatment history and relevant parameters were reviewed by the Multidisciplinary Team (MDT) including at least a radiation oncologist, a physicist, two radiologists, and experts in medical oncology and respiratory medicine. The study was performed in accordance with the Declaration of Helsinki of the World Medical Association and was approved by the Ethics Review Committees of The First Affiliated Hospital of Henan University of Science and Technology.

### Patients

Between June 2008 and June 2013, a total of -568 EC patients received DCRT with 3DCRT or IMRT at the First Affiliated Hospital of Henan University of Science and Technology. The clinical stages were classified according to the American Joint Committee on Cancer TNM Classification of Carcinoma of the Esophagus and Esophagogastric Junction (seventh edition, 2010). The diagnosis of ESCC was confirmed through gastrointestinal endoscopy and pathological biopsy. The histological type and grade of the disease were determined by experienced consultant pathologists and laboratory technicians.

### Case and control selection

#### Inclusion criteria for case

**Figure 2:**
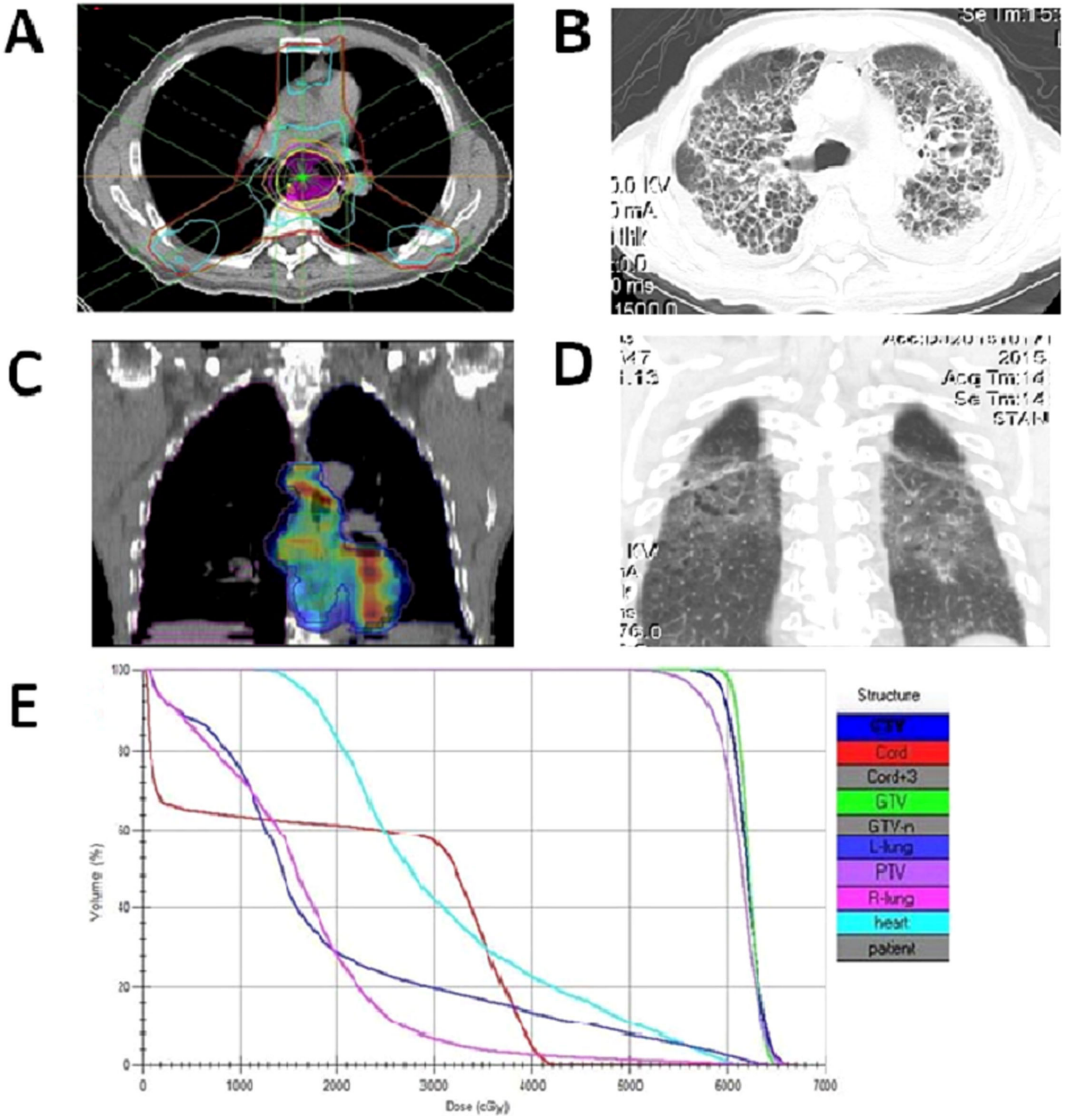
The dose distribution of the radiotherapy design and the AFRP CT images. **(A, B)** Images of patients with the 3DCRT radiotherapy dose distribution **(A)**, and a transverse view of the chest CT showing an interstitial pattern with traction bronchiectasis, opacities and a diffuse ground-glass pattern, bleb formation in the marginal areas, airspace consolidation and fibrosis in the bilateral lung fields **(B–D)** Images of patients with the IMRT radiotherapy dose distribution **(C)**, and the coronal view of the chest CT showing bilateral pulmonary volume with full diffuse ground-glass exudation **(D, E)** Detailed dose-volume distribution of one patient’s IMRT plan, V5s of the left lung and right lung are 86% and 88%, respectively. (3DCRT, three- dimensional conformal radiation therapy; IMRT, intensity-modulated radiation therapy).

AFRP was identified in patients who died from RP within 3 to 12 weeks after the beginning of DCRT (24). These patients presented with symptoms such as low- middle-grade fever, dry cough, and progressive dyspnea. Follow-up chest CT scans showed bilateral pulmonary distress with diffuse ground-glass exudation or diffuse irregular interlobular thickness and honeycombing, which indicated low-dose irradiation of nontarget organs at risk (**Figure 2**). Blood cultures, sputum cultures, and fungus cultures were all negative. Despite receiving standard treatment with antibiotics, steroid therapy, and supportive care, the patients succumbed to the progressive worsening of the disease.

Through the MDT experts reviewed all the ESCC patients receiving DCRT between June 2008 and June 2013, 22 patients died from AFRP within 6 months after receiving DCRT among the 568 patients mentioned. Five patients were excluded due to other complications: 3 with pulmonary embolism (PE) and 2 with cardiac failure (CF). There were 6 males and 11 females in the case group, all with clinical stages IIB-IIIC. None of the patients had received prior surgery, chemotherapy, or radiation.

#### Inclusion criteria for control

The inclusion criteria required that patients have stage IIB-IIIC ESCC at the time of initial diagnosis (according to Union for International Cancer Control TNM Classification, seventh edition) and had a survival period of more than 2 years after treatment. Patients who met any of the following conditions were excluded: previous surgery, chemotherapy, or radiation; pulmonary infections or diseases within 6 months prior to starting DCRT; or the use of protective reagents or steroid drugs to prevent or treat radiation damage during the DCRT process.

### Treatment

The prescribed doses for the planning target volume (PTV) ranged from 50 to 60 gray (Gy) delivered at 2 Gy per fraction which was in accordance with the China radiation oncology guideline at the time. The dose limit for normal lung tissue (more than 2 cm outside the target volume) was set at less than 40 Gy, with V40 (lung volumes receiving more than 40 Gy) limited to less than 15%. The regimens for DCRT included single-agent capecitabine, capecitabine in combination with oxaliplatin, and 5-FU plus cisplatin.

### Primary outcome

The primary outcome is to determine the risk factors for AFRP in lung volume-dose parameters, specifically V5, V20, V30 (lung volumes receiving more than 5 Gy, 20 Gy, or 30 Gy, respectively), and mean lung dose (MLD).

### Ethics statement

The study was performed in accordance with the Declaration of Helsinki of the World Medical Association and was approved by the Ethics Review Committees of The First Affiliated Hospital of Henan University of Science and Technology and written informed consent was obtained from each patient included in the study.

### Statistical analysis

Each case was matched to 3 selected controls based on the propensity score. Propensity score matching was estimated using a logistic model that included the following covariates: age, gender, smoking, ECOG performance status, chronic obstructive pulmonary disease (COPD), chemotherapy regimens, radiation method, PTV dose (Gy) and Radiation fields. These covariates were defined as gender (male/female), smoking history (yes/no), Eastern Cooperative Oncology Group (ECOG) performance status (0-1/≥2), and prior COPD were included as explanatory variables. (COPD, yes/no), chemotherapy regimen (single/dual agent (yes/no), radiation method (3DCRT/IMRT), and number of radiation fields. Positive smoking history was defined as smoking more than 1 cigarette per day for at least 6 months. Pre-existing COPD was assessed based on medical history, and there were no patients with COPD severity beyond grade 2. For each case, the three controls with the closest propensity scores were selected using nearest neighbor matching, in order to minimize confounding bias due to non-random group allocation.

One-to-three propensity score matching was conducted between case and control groups to minimize bias due to the non-random allocation among patients. Collinearity diagnostics were used to identify the multicollinearity among variables. All variables were included in the multivariable Cox regression model to further evaluate better prognostic predictors. Multivariable analyses were performed using the Cox proportional hazards model. Data analysis was performed using the statistical package SPSS 22.0 (IBM, California, United States). Odds ratios (ORs) were calculated and compared using the Mantel-Haenszen method, with a significance level of 0.05. Continuous variables were compared using t-tests for means, Mann-Whitney test for medians, and categorical variables were analyzed with chi-square or Fisher’s exact test. A Multivariate analysis was conducted using a logistic, stepwise method to screen the variables and a Variance Inflation Factor (VIF) to detect potential multicollinearity. VIF values were all below 2, indicating no significant multicollinearity. Both dependent and independent variables were included in the analysis based on complete case data. Additionally, a Cox proportional hazards regression model was used in the univariate analysis, with statistical significance set at p < 0.05.

## Results

Among 568 patients with ESCC who underwent DCRT at the First Affiliated Hospital of Henan University of Science and Technology from June 2008 to June 2013, a total of 22 patients died from the suspicious radiation pneumonitis, as confirmed by MDT group. Excluding 5 patients who had other complications: 3 with PE and 2 with CF, a final sample of 17 patients was included in the case group. A control group consisting of 51 patients was matched (**Figure 1**). DCRT was administered using a total dose of 50-60 Gy dose over 25-30 fractions and 5-FU based regimens. The demographic characteristics of the patients are shown in **Table 1**. No significant differences were observed between the two groups in terms of age, gender, smoking, propensity score, pre-existing COPD, and radiation fields after PSM matching (**Table 1**).

**Table 1:** Demographic characteristics (PSM-matched).

	RP Patients	Controls (n=51)	p*
	(n=17)		
Age (years) mean (standard deviation, SD	72 (5.445)	70 (7.949)	0.820
Gender, n (%)			1.000
Male	6 (8.8)	18 (26.5)	
Female	11 (16.2)	33 (48.5)	
Smoking, n (%) Yes	5 (7.4)	19 (27.9)	1.000
No	12 (17.6)	32 (47.1)	
ECOG performance status, n (%)			1.000
0-1	15 (22.1)	45 (66.2)	
2	2 (2.9)	6 (8.8)	
Pre-existing COPD, n (%) Yes	5 (7.4)	18 (26.5)	0.772
No	12 (17.6)	33 (48.5)	
Chemotherapy regimens, n (%)			1.000
5-FU**	7 (10.3)	21 (30.8)	
5-FU** + cisplatin	10 (14.7)	30 (44.1)	
Radiation method, n (%) IMRT	8 (11.8)	24 (35.3)	1.000
3DCRT	9 (13.2)	27 (39.7)	
PTV dose (Gy) median (range)	54.5 (50-60)	52 (50-60)	0.714
Radiation fields, n (%) ≤ 4	12 (17.6)	36 (52.9)	1.000
> 4	5 (7.4)	15 (22.1)	

** 5-FU or capecitabine was considered equal in this study. AFRP, acute fatal radiation pneumonitis; ECOG, Eastern Cooperative Oncology Group; COPD, chronic obstructive pulmonary disease; 5-FU, 5-fluorouracil; IMRT, intensity- modulated radiation therapy; 3DCRT, three-dimensional conformal radiation therapy; PTV, planning target volume.

**Figure 3:**
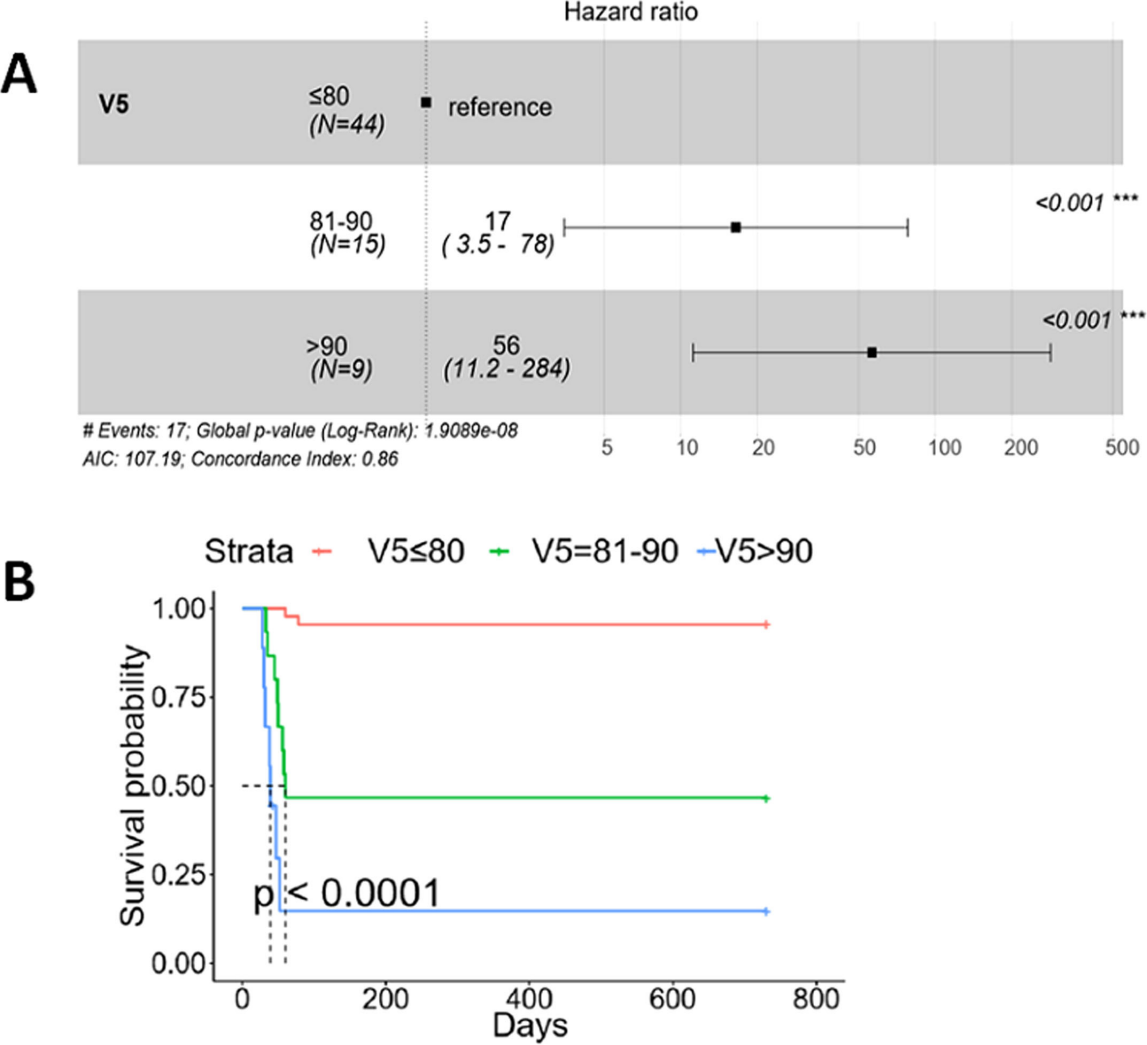
V5 contributing to the susceptibility of AFRP and overall survival. Different levels of V5 contributing to the susceptibility of AFRP and overall survival were analyzed using a Cox proportional hazards regression model. **(A)** Compared to V5 < 80%, both V5 = 81-90% and V5 > 90% significantly increased the susceptibility of AFRP (HR: 17, 95% CI: 3.5-78, *p* < 0.001; HR: 56, 95% CI: 11.2-284, *p* < 0.001, respectively). **(B)** Compared to V5 < 80%, EC patients receiving CRT with either V5 = 81-90% or V5 > 90% had worse overall survival (*p* < 0.0001). (AFRP, acute fatal radiation pneumonitis; CRT, concurrent chemoradiotherapy.

Comparing the values for the control group, the cases group exhibited significantly higher median values for V5 and MLD (88.39% versus 65.04%, *p*=0.005, and 17.32 Gy versus 14.38 Gy, *p* = 0.005, respectively) (**Table 2**). This suggests that EC patients undergoing DCRT may have an elevated risk of AFRP with elevated V5 and MLD values. Through conditional logistic regression analysis, it was determined that V5 > 60%, V20 > 25%, and MLD > 15 Gy were independent risk factors for AFRP (**Table 3**). Cox proportional hazards regression analysis revealed that V5 > 80% significantly increased the susceptibility of AFRP (V5 = 81-90%: hazard ratio (HR): 17 (95% CI: 3.5-78), *p* < 0.001; V5 > 90%: HR: 56 (95% CI: 11.2-284), *p <*0.001) (**Figure 3A**). This further corroborates the hypothesis that an elevated V5 is associated with an increased risk of AFRP occurrence. Additionally, as V5 rises, the impact on patient survival may be more pronounced (*p* < 0.0001) (**Figure 3B**).

**Table 2:** Lung dose-volume parameters.

Parameters	RP Patients (n=17)	Controls (n=51)	P^*^
	Median (range)		
V5 (%)	88.39 (72-99.56)	65·045 (27.1-92.9)	0·005
V20 (%)	32.4 (22.83-42.13)	23.775 (1.95-31.87)	0·05
V30 (%)	13.69 (5-16.88)	9.06 (2.84-16.88)	0·700
MLD (Gy)	17.325 (15.08-19.78)	14.38 (3.7-17.97)	0·005

MLD, mean lung dose.

**Table 3:** The odds ratio of lung dose-volume parameters.

	OR (95% CI)	*P*
V5		
≤ 60%	1	
> 60%	13.818 (1.992-96.322)	0.001
V20		
≤ 25%	1	
> 25%	2.159 (1.234-3.778)	0.025
V30		
≤ 15%	1	
> 15%	1.727 (0.536-5.566)	0.627
MLD		
≤ 15Gy	1	
> 15Gy	2.375 (1.402-4.024)	0.005

MLD, mean lung dose.

## Discussion

CRT with 5-FU and/or cisplatin regimens has been shown to improve overall survival in patients with EC. At follow-up of at least 5 years, the Radiation Therapeutic Oncology Group Trial 85-01 reported an overall survival rate of 26% for EC patients who received CRT compared to 0% for those who received radiotherapy alone. Grade 3 or higher toxicity occurred in 50% of EC patients, with only 1 patient experiencing grade 4 RP out of a total of 117 patients in the CRT group (6, 25). Another study showed that DCRT resulted in a very high complete remission rate (93%) and moderate radiation toxicity, with an incidence rate of grade 2 RP at 18% (26). In a study of Chinese EC patients, the incidence of severe RP was reported in five out of thirteen patients treated with CRT (27). Similarly, in our study, we observed a higher incidence of AFRP in a short time period, with 17 cases of grade 5 RP out of 568 EC patients receiving CRT. It is worthwhile to understand the contributing factors to such severe cases.

Several factors have been identified as high-risk factors for RP, such as poor performance status, elderly age, history of smoking, chronic pulmonary disease, gender, tumor location, tumor volume and lung dosimetric parameters. A meta-analysis of 31 independent studies comprising patients with diverse thoracic malignancies (lung, breast, and esophageal cancer) revealed that older age and pre-existing lung disease were associated with an increased risk of RP (19–21). The location of the tumor has been identified as a risk factor for RP in numerous studies (22), Furthermore, it appears that an increase in tumor volume is associated with an elevated risk of developing RP (23). Adequate control of lung radiation parameters is crucial in preventing RP, especially in selecting suitable patients based on other factors. High radiation doses and large fractionation doses have been associated with higher incidence of RP in some studies. A study reported a 4% incidence of grade 3 or higher RP, with 2% of EC patients experiencing fatal RP when receiving CRT at doses of 60-66 Gy (28). Another study showed that pulmonary complications significantly increased when V10 exceeded 40% (29). Other volume-dose histogram parameters, such as V15 ≥ 30% and V20 ≥ 20%, have also been linked to 32% to 35% of pulmonary complications (30). More recent evidence suggests that an absolute volume of lung-spared doses of > 5 Gy is correlated with RP (31). The American National Comprehensive Cancer Network guidelines recommend limiting V5 and V20 to less than 50% and 25%, respectively, in EC patients receiving radiotherapy (32). A study attributed the high incidence of fatal pneumonitis after IMRT for mesothelioma to large volumes of lungs receiving low doses, especially V5 > 90% (33). In this study, dosimetric parameters were limited to V45< 15%, V30 < 20%, and V20 < 40% for patients receiving CRT, but there was no specific limitations for V5. The median V5 values were 88.39% in the case group and 65.04% in the control group. When V5 > 80%, there was a significant increase in the susceptibility of AFRP and a worse overall survival. For this result, we analyzed the reasons as follows: The patients included in the study were treated between 2008 and 2013. At that time, the control of lung V5 was still in the stage of research and gradual acceptance. Some studies had suggested that reducing V5 could decrease the incidence of radiation pneumonitis, leading to increasing clinical attention to this parameter. However, V5 had not yet been strictly standardized as a global guideline indicator before 2013. In recent years, V5 has been limited to ≤50% according to NCCN guideline which represents a significant advancement and markedly reducing the incidence of radiation pneumonitis for esophageal cancer patients requiring DCRT (34). In our previous study, a prospective phase III clinical study on DCRT for locally advanced esophageal cancer, we implemented a strategy to limit to V5 ≤ 50%, and performed chest CT scans when patients receiving radiation with 40 Gy to assess the extent of radiation-induced lung damage in advance. This approach yielded positive results, with the incidence of grade 3-4 radiation pneumonitis being 14%, and no cases of fatal radiation-induced lung injury (35).These findings support that low-dose volume may play an important role in preventing severe radiation lung injury and treatment-related death.

IMRT is a more advanced radiation technology that has been increasingly used in China. Compared to 3DCRT, IMRT allows for better visualization of anatomical structures and improved target delineation for dose sparing of normal tissues. However, 3DCRT achieves accurate delivery of high-dose radiation to the target area by adjusting the shape of non-coplanar high-energy beams, while greatly reducing radiation exposure to normal tissues. In contrast, IMRT adjusts the beam intensity across the irradiation field, ensuring that the high-dose region conforms to the shape of the tumor and that the dose is uniformly distributed within the target area. Based on the above theoretical background, when esophageal cancer patients undergo radiotherapy, the use of IMRT may result in more lung tissue receiving radiation compared to 3DCRT. In a study, 3DCRT-treated EC patients had a significantly higher mortality rate (72.6% versus 52.9%) and higher local-regional recurrence compared to IMRT-treated patients, with an increased incidence of cardiac death in the 3DCRT group (36). Another study also reported a significantly lower incidence of RP in EC patients treated with IMRT compared to those treated with 3DCRT (37). However, in our study, the incidence of AFRP in the IMRT group (2.9%, 8/274) was higher than in the 3DCRT group (1.5%, 9/294), although it was not statistically significant. There was no difference between the IMRT and 3DCRT groups in age, gender, performance status, and basic lung function. A study found that V20 was larger in the IMRT group compared to the 3DCRT group, and that V20 (> 15%) and V30 (> 20%) were associated with increased incidence of chronic and acute pneumonitis, respectively (38). Our findings are consistent with this study.

We also observed a higher incidence of AFRP in female patients compared to male patients (7.7% versus 0.8%, respectively) in this study. This may be explained by the smaller lung capacity in women, but they may also exhibit a stronger immune response. Therefore, under certain conditions, female patients may be more susceptible to acute respiratory failure and death. This is in accordance with the study (31), which identified volume of lung spared from doses of 5 Gy or higher as the only independent predictive factor for postoperative pulmonary complications in EC patients treated with CRT followed by surgery. They found that smaller volumes of lung receiving doses less than 5 Gy were associated with higher incidence of postoperative pulmonary complications, and patient sex was associated with the incidence of these complications in univariate analyses. However, more research is needed in the future. To reduce the risk of AFRP, attention should be given not only to the dose volume histogram of the lung but also to the total lung volume and non-irradiated lung volume during treatment planning.

However, the study has several limitations. The retrospective nature of the study inherently brings selection biases, despite the use of propensity score matching (PSM), and the lack of dynamic monitoring data during radiotherapy limits a more detailed understanding of the temporal relationship between lung function changes and dose-volume parameters. Other potentially relevant factors, such as genetic polymorphisms and comprehensive nutritional status, were not considered, which could have important interactions with the studied parameters. Further research is planned to explore the identified risk factors, the long-term outcomes of patients with different risk profiles, and to develop predictive models that incorporate multiple factors to improve risk stratification.

## Conclusion

V5 > 60%, V20 > 25%, and MLD > 15 Gy are the key risk factors for developing AFRP among EC patients receiving DCRT, and V5 > 80% may serve as a powerful predictor of mortality risk.

## Data Availability

The original contributions presented in the study are included in the article/supplementary material. Further inquiries can be directed to the corresponding authors.
